# Association between Non-Suicidal Self-Injuries and Suicide Attempts in Chinese Adolescents and College Students: A Cross-Section Study

**DOI:** 10.1371/journal.pone.0017977

**Published:** 2011-04-08

**Authors:** Jie Tang, Yizhen Yu, Yu Wu, Yukai Du, Ying Ma, Huiping Zhu, Ping Zhang, Zhuoya Liu

**Affiliations:** 1 Department of Child, Adolescence and Woman Health Care, School of Public Health, Tongji Medical College, Huazhong University of Science and Technology, Wuhan, Hubei, China; 2 Guangzhou Women and Children's Medical Center, Guangzhou, China; Pennsylvania State University, United States of America

## Abstract

**Purpose:**

This study examined the association between non-suicidal self-injury (NSSI) and suicide attempts among Chinese adolescents and college students.

**Methods:**

A total sample of 2013 Chinese students were randomly selected from five schools in Wuhan, China, including 1101 boys and 912 girls with the age ranging between 10 and 24 years. NSSI, suicidal ideation, suicide attempts and depressive symptoms were measured by self-rated questionnaires. Self-reported suicide attempts were regressed on suicidal ideation and NSSI, controlling for participants' depressive symptoms, and demographic characteristics.

**Results:**

The self-reported prevalence rates of NSSI, suicidal ideation, suicide attempts were 15.5%, 8.8%, and 3.5%, respectively. Logistic regression analyses indicated that NSSI was significantly associated with self-reported suicide attempts. Analyses examining the conditional association of NSSI and suicidal ideation with self-reported suicide attempts revealed that NSSI was significantly associated with greater risk of suicide attempts in those not reporting suicidal ideation than those reporting suicidal ideation in the past year.

**Conclusions:**

These findings highlight the importance of NSSI as a potentially independent risk factor for suicide attempts among Chinese/Han adolescents and college students.

## Introduction

Suicide is one of the leading causes of death among adolescents and young adults around the world [1], [2]. In the United States, it has been reported that suicide is the third leading cause of deaths for people aged 10 to 24 years, that about one third of adolescents reported having experienced suicidal ideation during their lifetime, and that suicide attempts are made by about one tenth [3]. In China, suicide is the fifth most important cause of deaths in the general population, and the first leading cause among the persons aged 15 to 34 years [4].

One important characteristic of attempted and completed suicides is the extent to which the act was planned. Unplanned acts of suicide involve ‘little preparation or premeditation’ [5]. Planned and unplanned suicide attempts differ in many aspects. Firstly, planned suicide attempts have generally been associated with higher levels of depression, hopelessness, and lethality [6], and planned suicide attempts usually have suicidal ideation long enough, while unplanned suicide attempts are more common among males and those with greater aggressiveness [7], in which suicidal ideation usually can not be detected at all prior to their attempts. In addition, unplanned suicidal behaviors among youths may be more likely to occur following stressful life events [8]. Secondly, since planned suicide attempts are generally associated with mental disorders (i.e., depression), those at risk for planned acts may be easily identifiable, while unplanned acts may be less easily identifiable due to there are less visibly mental disorders, which may make the prevention efforts focused on identifying individuals exhibiting the signs or symptoms of suicidal behavior difficult [9]. Furthermore, recent study have found a trend of increasing prevalence of suicide attempts with decreasing prevalence of planned acts and suicidal ideation among youths [10], suggesting that the rate of suicide attempts in the absence of suicidal ideation may be increasing.

Over the last decade, to predict and prevent suicide, researches have focused on risk factors related to suicide attempts. Most of previous studies aimed at identifying risk factors for suicide attempts have focused on the importance of prior mental disorders, and these studies have shown that mental disorders, especially depressive disorders, are among the strongest risk factors for suicide attempts [11], [12]. Despite of this knowledge and related prevention measures put into place, problems remain. First, there was no mental disorder that was uniquely predictive of suicide attempts. Virtually, all mental disorders, such as depression, panic disorder, and anxiety disorder, are associated with an increased risk of suicide attempts [13], [14]. Second, little is known about the extent to which mental disorders predict suicide attempts beyond their association with suicidal ideation. Recent studies have found that although mental disorders are strongly predictive of suicidal ideation, they are less useful in predicting who with suicidal ideation will go on to have suicide attempts [15], [16]. Furthermore, there seem more and more suicide attempts without suicidal ideation among adolescents and youths [10].

According to the interpersonal-psychological theory of suicidal behaviors, people will not die by suicide unless one has both the desire to die and the willingness to die [17]. The theory asserts that people hold perceived burdensomeness and a sense of low belongingness or social alienation for long enough will desires to die, namely suicidal ideation. However, self-preservation is powerful enough instinct that few of us can overcome by the force of will, and few of us have developed a fearlessness of pain, injury, and death, which can be acquired through a process of repeatedly experiencing painful, for instances, non-suicidal self-injury (NSSI), which is the most commonly described as deliberate direct destruction of alteration of body tissue without conscious suicidal intent [18].

However, the extant studies that have investigated the relationship between NSSI and suicidal behaviors have showed no consistency. In the west, some studies showed that there was a strong correlation between suicidal behaviors and NSSI [19], and that as the severity of NSSI accelerated, the severity of suicidal behaviors increased as well [20]. While other studies found that this relationship may not exist because NSSI was to reduce negative affect or emotion but not to cause suicidal behaviors [21], [22]. In China, these kinds of studies were little reported.

The current study was designed to investigate the association of NSSI and suicide attempts in Chinese adolescents and college students. Based on the literature review, we hypothesized that NSSI would be associated with suicide attempts, even after taking into account depressive symptoms, demographic characteristics and the association would be modified by suicidal ideation.

## Methods

### Study Population

This study was conducted in Wuhan, a metropolitan city located in the center of China. A multistage cluster sampling was conducted to generate a diverse sample. First, two districts that represented the general level of socioeconomic status were selected from 15 districts in Wuhan [23]. Then, in the selected two districts, two junior high schools, two high schools and a college were selected randomly. In each of the selected schools, one class or two were randomly selected for each grade. In the selected classes, all the students were eligible for inclusion in the study except students who have severe mental disorders (depressive disorder, anxiety disorders, obsession, schizophrenia, etc) and severe parenchymal disease (heart disease, diabetes, hepatitis, pulmonary tuberculosis, etc). Overall, 2409 students were selected and a consent letter was sent to the students (college students) or their parents. Of these study subjects, 231 refused to participate in the study and 44 were absent from the school at the time of survey; therefore, the questionnaire was completed by 2134 students. By an initial screening based on the completeness of the questionnaires, 121 students were excluded due to incomplete questionnaires. Thus, the final study population in this study consisted of 2013 students, 1101 boys and 912 girls, with a mean age of 15.6 (SD = 2.8) years, ranging between 10 and 24 years. The actual ratio of participants was 94.3% (2013/2134), all participants were of the Han nationality and none of them were from psychological education school. The Ethical Committee of the Medical Association of Tongji Medical College of Huazhong University of Science and technology approved the study, and each participant has obtained a written informed consent.

The survey was anonymous, and it was completed in more private environment.

Data collection was carried out in March 2007 by a group of trained investigators, who explained the purpose and procedures of the study on the spot. Before the students completed the self-reported questionnaire, they were told that the questionnaire did not represent a test and that there were no correct or incorrect answers and we have promised each participant that their answers will be kept confidential and the data will only be used for scientific research. The same written announcements were printed in the front of the questionnaire. The emphasis was placed on answering the questions honestly and accurately.

### Instrument for Non-suicidal Self-Injury

The Functional Assessment of Self-Mutilation (FASM) [24] was designed to assess NSSI of the participants over the previous 12 months. The FASM is a semi-structured clinical interview that evaluates behavioral functions and the frequency of different methods of NSSI. It has demonstrated acceptable psychometric properties within adolescent samples [25]. The FASM contains ten behaviors yielding two factors: ‘moderate/severe NSSI’ and ‘minor NSSI’ [24]. Previous research has described the factor structure, providing a support for the reliability and validity of the FASM within the current sample [26]. In our study, the FASM was designed as a self-report measure, and only the frequency of different methods of NSSI was measured.

### Suicidal ideation, suicide attempt

Suicidal ideation was measured by the question, “During the past 12 months, did you ever seriously consider attempting suicide?” Response options were “yes” or “no”. Suicide attempt was measured by the question, “During the past 12 months, how many times did you actually attempt suicide?” For this item, responses were fallen into 2 categories, 0 times vs. 1 or more times. These questions have demonstrated substantial reliability. The 2-week test-retest was 83.8% for suicidal ideation and 76.4% for suicide attempts [27].

### Depressive Symptoms

Respondents completed the 20-item on the Center for Epidemiological Studies Depression Scale (CES-D), which has been used extensively in studies of adolescents [28]. Items are rated from 0 to 3, with higher scores indicating greater severity of depressive symptoms. In the present survey, the CES-D had a very good internal consistency (α = 0.85).

### Statistical Analysis

Descriptive statistics (mean, standard deviation, and percentage) were calculated to reflect the background characteristics of the study sample. Chi-square tests and Student *t*-tests were used to compare frequencies and continuous data, respectively. The probability of attempting suicide within the past year was estimated in logistic regressions that included predictors of depressive symptoms, suicidal ideation, NSSI, and demographic controls. Demographic controls included sex, age, learning phases (middle high and college) and parents' marital status. A product term representing the interactions between suicidal ideation and NSSI was also included in subsequent models to test for differences in the association between NSSI and self-reported suicide attempts among youths with and without suicidal ideation. As a multistage cluster sampling was applied in the study, sampling weight was considered for all the Logistic regressions, the sampling weight is a product of reciprocal of the probability of multiple sampling. Significance level was set at 0.05, and all tests were two sided. Statistical analyses were conducted using Statistical Package for Social Sciences software (SPSS for Windows 15.0, SPSS Inc., Chicago, IL).

## Results

### Overview

Simple Chi-square tests indicated that NSSI was significantly associated with suicide attempts. Among participants who did not report suicidal ideation, NSSI was also associated with suicide attempts. This association was further replicated in a Binary Logistic Regression analysis. NSSI was associated with increased risk for suicide attempts, controlling for demographic characteristics, depressive symptoms and suicidal ideation. Additionally, NSSI was associated with an increase in the risk of suicide attempt among those who did not report suicidal ideation compared with those who did report suicidal ideation.

The demographic characteristic and prevalence rates of risk behaviors of the sample are showed in Table 1. Self-reported one-year prevalence rate of suicidal ideation and suicide attempts were 8.8% and 3.5%, respectively. Significant sex differences were found in one-year prevalence of suicidal ideation (χ2 = 10.43, *p* = 0.001) and suicide attempts (χ2 = 6.32, *p* = 0.01). Self-reported one-year prevalence rate of NSSI was 15.5%, with 10.5% and 5.0% of adolescents engaged in “minor” NSSI, and “moderate/severe” NSSI, respectively. Males were more likely to be engaged in NSSI (both “minor” and “moderate/severe” NNSI) than females (χ2 = 9.75, *p* = 0.008).

**Table 1:** Demographic characteristics and prevalence of risk behaviors of the study population.

	Boys (n = 1101)	Girls (n = 912)	Total (n = 2013)
Age, n (%)			
10∼12 yr	188 (17.1)	152 (16.7)	340 (16.9)
13∼15 yr	403 (36.6)	304 (33.3)	707(35.1)
16∼18 yr	313 (28.4)	253 (27.7)	566(28.1)
19∼24 yr	197 (17.9)	203 (22.3)	400(19.9)
Depressive symptoms (Mean, SD)	8.1±6.6	10.1±6.4	9.0±6.6
NSSI, n (%)			
Non-NSSI	906(82.3)	794(87.1)	1700 (84.5)
Minor NSSI^a^	137(12.4)	76(8.3)	213 (10.6)
Moderate/severe NSSI^b^	58(5.3)	42(4.6)	100 (5.0)
Suicide ideation during the last year (n, %)	86(7.8)	92(10.1)	178 (8.8)
Suicide attempts during the last year (n, %)	28(2.5)	42(4.5)	70 (3.5)

Note: NSSI = Non-suicide self-injury;

a Minor NSSI included hitting self, pulling hair, biting self, inserting objects under nails or skin, picking at a wound, and picking areas to draw blood.

b Moderate/severe NSSI included cutting/carving, burning, self-tattooing, scraping, and erasing skin.

The overlap between suicide attempts and NSSI among the adolescents in the sample is presented in Table 2. NSSI was significantly associated with self-reported suicide attempts (χ2 = 114.53, *p*<0 .001). Almost 22.0% of the subjects who were engaged in “moderate/severe” NSSI and 5.6% of the adolescents who were engaged in “minor” NSSI reported suicide attempts in the last year, while the prevalence rate of suicide attempt was only 2.1% of the adolescents who reported non-NSSI. Additionally, suicidal ideation was strongly associated with self-reported suicide attempts, 27.4% of respondents who reported thinking about suicide, compared with 1.4% of those who did not reported suicide attempts (χ2 = 613.64, *p*<0.001). Odd ratios for NSSI stratified by suicide ideation are presented in Table 3. OR in subjects who did not report suicide ideation indicated that engaged in NSSI in the last year was associated with more than fivefold increase in risk of self-reported suicide attempts (OR = 5.66, 95% CI = 2.59, 12.38), while in subjects who reported suicide ideation, engaged in NSSI was associated with twofold increase in risk of self-reported suicide attempts (OR = 2.27, 95% CI = 1.13, 4.55).

**Table 2:** Numbers and column percents for suicide attempts within reported NSSI and ideation.

		NSSI			Suicide ideation		Total
		Non-NSSI	Minor NSSI	Moderate/severe NSSI	No	Yes	
Suicide attempts	Yes	36 (2.1%)	12 (5.6%)	22 (22.0%)	26 (1.4%)	44 (24.7%)	70 (3.5%)
	No	1664 (97.9%)	201 (94.4%)	78 (78.0%)	1829 (99.7%)	134 (75.3%)	1943 (96.5%)
	Total	1700 (84.5%)	213 (10.6%)	100 (5.0%)	1835 (91.2%)	178 (8.8%)	2013 (100%)

Note: NSSI = Non-suicide self-injury;

**Table 3:** The odd ratio for NSSI stratified by suicide ideation.

Suicide ideation	Suicide attempts	NSSI		OR	95%CI for OR	
		No	Yes		Lower	Upper
No	No	1571	238	5.66	2.59	12.4
	Yes	14	12			
Yes	No	93	41	2.3	1.1	4.5
	Yes	22	22			

To examine these associations in a multivariate context, after considering the sampling weight, a Binary Logistic Regression analysis was performed, in which self-reported suicide attempts were regressed on suicidal ideation and the NSSI, along with controlling for the participants' levels of depressive symptoms and demographic characteristics. Results from the main effect model are showed in Table 4 (Model I). Obviously, reporting suicidal ideation in the past year dramatically increased the risk of suicide attempts (OR [odds ratio] = e^2.78^ = 16.18, 95% CI [confidence interval] = 9.36, 27.97). Results also indicated that NSSI was significantly associated with increased risk for self-reported suicide attempts (OR = e^0.85^ = 2.35, 95% CI = 1.67, 3.29) after controlling for depressive symptoms, suicidal ideation, and other demographic characteristics. NSSI conveyed more than a 135% increase in the risk of self-reported attempts in the participants.

**Table 4:** Logistic regression models predicting suicide attempts.

	Model I		Model II		Model III		Model IV	
	OR	95% CI for OR	OR	95% CI for OR	OR	95% CI for OR	OR	95% CI for OR
Female vs. male	1.60*	1.13∼2.08	1.61*	1.13∼2.09	1.96*	1.02∼3.72	1.59*	**1.23∼1.70**
Age in years	1.06*	1.03∼1.09	1.06*	1.01A∼1.11	1.06*	1.01∼1.11	1.10*	**1.03∼1.18**
Suicide ideation (yes vs. no)	16.18*	9.36∼27.97	23.58*	11.81∼47.07	19.23*	10.49∼35.24	22.59*	**11.49∼44.4**
NSSI (yes vs. no)	2.35*	1.67∼3.29	5.91*	2.08∼16.83	3.68*	1.72∼3.87	5.22*	**1.98∼13.79**
Depressive symptoms scores	1.04*	1.01∼1.08	1.04*	1.02∼1.08	1.05*	1.01∼1.08	1.05*	**1.01∼1.08**
Suicide ideation*NSSI	—	—	0.56*	0.53∼0.59	—	—	—	**—**
Suicide ideation*NSSI*Gender	—	—	—	—	0.83	0.63∼1.11	—	**—**
Suicide ideation*NSSI*Age	—	—	—	—	—	—	0.97	**0.93∼1.01**
Constant	−9.52	—	−10.20	—	−10.01	—	−10.62	—

Note: NSSI = Non-suicide self-injury; CI = Confidence Interval. *p<0.05, n = 2013.

To test whether the association between NSSI and suicide attempts differed among those who had and had not reported suicidal ideation, the interaction of suicidal ideation with NSSI was added separately to the regression equation presented in Table 4 (Model II). The coefficient for the interaction of suicidal ideation and NSSI was significant and negative (β = −0.59, SE = 0.18), indicating that NSSI was associated with a higher risk of suicide attempts among those who did not report suicidal ideation. OR calculated from the coefficients in this model indicated that engaged in NSSI in the last year was associated with nearly sixfold increase in risk of self-reported suicide attempts among those who did not report suicidal ideation(OR = e^1.77^ = 5.91, 95% CI = 2.08, 16.83), but was associated with almost threefold increase among those who did report suicidal ideation (OR = e^1.77-0.59^ = 3.25).

Additional models were estimated to test whether the association of NSSI and suicidal ideation on suicide attempts differed among demographic groups defined by sex and age. None of the three-way interaction terms estimated in these models achieved statistical significance at the 0.05 level (Model III and Model IV).

## Discussion

In this study, we investigated association between NSSI and suicide attempts based on the interpersonal-psychological theory of suicidal behavior proposed by Joiner in 2005 [17]. The results revealed that NSSI was associated with suicide attempts after adjustment for sex, age, depressive symptoms and suicidal ideation. The results supported one aspect of the interpersonal-psychological theory and were consistent with previous findings on the association between NSSI and suicidal behaviors [20], [29].

During the last decade, there has been growing concern about youth suicide, which led to growing research of the prevalence, correlates, and etiology of suicidal behaviors. In our study, the one-year suicidal ideation and attempts rates were 8.8% and 3.5%, respectively, which were similar to the previous studies conducted in China [30].

As for risk factors for suicidal behaviors, previous studies all over the world have suggested that suicide attempts and suicide have the same risk factors, which are a result of the interaction of different risk factors from family, social, environment and personally [31]. With the reference to family perspective, different family environments are postulated to be related to adolescent suicidal behavior, such as communication disturbances and excessive secretiveness [32], family conflict [33], sexual or physical abuse [34], family neglect. Other social and environmental risk factors include homeless, difficulties in school, social isolation, and presence of stressful life events. Personal mental health problems that predispose to suicidal behaviors include depression, bipolar disorder, substance abuse or dependence, post traumatic stress disorder, and panic attacks [35]. Although most previous studies aimed at to identify the risk factors of suicidal behaviors, problems still exist, because so many risk factors are related to suicidal behavior that it seems everyone is possible to execute suicidal behaviors. Hence, the real problem we need to consider is that why so many people have risk factors, and yet why did only a few experienced suicide attempts and/or suicide? The interpersonal-psychological theory of suicidal behaviors suggests that people will not die by suicide unless one has both the desire to die and the willingness to die [17]. Therefore, those risk factors mentioned above may instill a suicidal ideation, while they are not sufficient to ensure that the desire will lead to a suicide attempts and suicide. For suicide to occur, other elements must be present at the same time, namely, the acquired ability for suicide. Because self-preservation is a human instinct, body is generally not designed to cooperate with its own demise, and suicide attempts happened only when self-preservation is beaten up. The ability for suicide seems to play a more important role in youth suicidal behaviors, because adolescents may be more impulsive and may have a different time perspective than adults, and may focus more on proximal consequences of behaviors than more distant goals when making decisions [36], under this condition, suicidal ideation is hardly to detect. In our study, the subjects who attempted suicide but not have suicidal ideation accounted for 0.3%.

NSSI was usually not considered as an illness, which may explain the rising prevalence rate of NSSI all over the world [37]. Prior studies conducted in the west reported that the prevalence rates of NSSI ranged between 6.2% and 17% in adolescents [37]. In our study, the prevalence rate of NSSI was 15.5%, with 10.5% and 5.0% of adolescents engaged in “minor” NSSI and “moderate/severe” NSSI, respectively, which was consistent with the previous findings conducted in China and the west [38], [39].

Numerous functions of NSSI are indicated in the clinical literature, including the need to relieve negative emotions, such as anxiety, guilt, loneliness, alienation or self-hatred; to relieve unpleasant thoughts or feelings; to release anger, tension or emotional pain; to provide a sense of security or control; to punish self; to set boundaries with others; to end depersonalization or derealization, flashbacks or racing thoughts [40]; for this perspective, aversive emotions (anger, depression, loneliness and frustration) were reduced during and following NSSI, while emotions deemed as positive (relief) and self-conscious (guilt, shame ,disgust) increased following NSSI [41]. However, some investigators suggested that as much as 40% of those who were engage in NSSI had thoughts about suicide while inflicting the injury [42], and approximately 50% to 85% of people who injured themselves had attempted suicide at least once during their lifetime [43]. In our study, there were 10.9% of those who were engaged in NSSI had attempted suicide during the last 12 months, which is significantly higher than that of people who were engaged in no NSSI. Furthermore, subjects who were engaged “moderate/severe NSSI” were more likely than those who were engaged “minor NSSI” to attempted suicide (22.0% vs. 5.6%). This was similar to the recent findings reported by Whitlock and colleagues, who examined self-injury correlates in 2101 university students [44], in which the results indicated that as the severity of self-injury accelerated, the severity of suicidal behaviors increased as well. In additional, in the present study, NSSI was significantly associated with increased risk for self-reported suicide attempts (OR = e^0.85^ = 2.35, 95% CI = 1.67, 3.29), after controlling for depressive symptoms, suicidal ideation, and other demographic characteristics. What's more, NSSI was associated with a higher risk of suicide attempts among those who did not report suicidal ideation,which suggests that NSSI may promote increased impulsivity and aggressiveness, leading to suicide attempts and suicide among non-ideators.

As a method of acquiring ability to enact suicide, the basis for this proposition rests primarily on the opponent-process theory, which suggests that with repeated exposure to an affective stimulus, the reaction to that stimulus shifts over time such that the stimulus loses its ability to elicit the original response and, instead, the opposite response is strengthened [45]. According to this, NSSI can be viewed as a progress of habituation or tolerance for pain and a sense of fearlessness in the face of death. Mark AI and colleagues found chronic pain was a potentially independent risk factors suicide [46], which also can be explained by the opponent-process theory. However, Nock et al. offered an alternative to Joiner's theory based off their findings that individuals with more extensive histories of NSSI were more likely to attempt suicide but the frequency of their NSSI was not linked to suicidality [47]. They suggested that some who self-injure do not necessarily habituate to the pain associated with the behavior, but simply fail to feel pain even when they first begin self-injuring. In the present study, most students (account for 64.7%) who reported suicide attempts engaged in NSSI once or twice during the last year. In spite of this, a simply description of the NSSI frequency may not explicit how to overcome pain by engaged in NSSI, because data on the feelings of the first NSSI and the total frequency of NSSI before suicide attempts occurred were not collected in the present study.

The present study must be interpreted in light of several limitations. First, although the study achieved a relative large sample size, the age span of the sample range too large, which may be possible selection bias exists. Some younger students (10∼12 years) may be not with the same capacity to fully understand the self-rated questionnaire as elder students (19∼24 years), though a group of investigators were trained to explain the question on the spot of survey to help the subjects to understand the self-rated questionnaire. Each of these factors limits the generality of the results.

Second, these data are based on retrospective self-reported of the occurrence of suicidal behaviors, which introduce potential problems with underreporting and bias recall. Nonetheless, the self-reported suicidal ideation and attempts in our study were similar to those found in another independent study conducted on a sample of children of the same age range in China [30]. In addition, it is possible that the items related to NSSI may be erroneously reported, as autobiographical memory is constructive and not productive, and it is prone to errors and illusions [48].

Third, we did not consider some other factors when we assessed the association between NSSI and suicide attempts, including student's personality, family conditions (parents' occupation, attitude, relationship with their child, etc) and so on, which might/could led to an underestimate of strength of associations between NSSI and suicide attempts. Therefore, the investigation of these and other factors remain key directions for future research.

Fourth, this study was cross-sectional, and causal processes linking NSSI to suicide attempts and/or suicide must be inferred and cannot be tested in the data. Therefore, the findings should be validated in larger, prospective studies.
